# SPLIT-PIN software enabling confocal and super-resolution imaging with a virtually closed pinhole

**DOI:** 10.1038/s41598-023-29951-9

**Published:** 2023-02-15

**Authors:** Elisabetta Di Franco, Angelita Costantino, Elena Cerutti, Morgana D’Amico, Anna P. Privitera, Paolo Bianchini, Giuseppe Vicidomini, Massimo Gulisano, Alberto Diaspro, Luca Lanzanò

**Affiliations:** 1 Department of Physics and Astronomy “Ettore Majorana”, University of Catania, Via S. Sofia, 64, 95123 Catania, Italy; 2 Laboratory of Synthetic and Systems Biology, Department of Drug and Health Sciences, University of Catania, Catania, Italy; 3 Molecular Preclinical and Translational Imaging Research Centre-IMPRonTe, University of Catania, Catania, Italy; 4 Interuniversity Consortium for Biotechnology (CIB), Trieste, Italy; 5 Nanoscopy, CHT Erzelli, Istituto Italiano di Tecnologia, Genoa, Italy; 6 Molecular Microscopy and Spectroscopy, CHT Erzelli, Istituto Italiano di Tecnologia, Genoa, Italy; 7 DIFILAB, Department of Physics, University of Genoa, Genoa, Italy

**Keywords:** Biological fluorescence, Nanoscale biophysics, Confocal microscopy, Super-resolution microscopy

## Abstract

In point-scanning microscopy, optical sectioning is achieved using a small aperture placed in front of the detector, i.e. the detection pinhole, which rejects the out-of-focus background. The maximum level of optical sectioning is theoretically obtained for the minimum size of the pinhole aperture, but this is normally prevented by the dramatic reduction of the detected signal when the pinhole is closed, leading to a compromise between axial resolution and signal-to-noise ratio. We have recently demonstrated that, instead of closing the pinhole, one can reach a similar level of optical sectioning by tuning the pinhole size in a confocal microscope and by analyzing the resulting image series. The method, consisting in the application of the separation of photons by lifetime tuning (SPLIT) algorithm to series of images acquired with tunable pinhole size, is called SPLIT-pinhole (SPLIT-PIN). Here, we share and describe a SPLIT-PIN software for the processing of series of images acquired at tunable pinhole size, which generates images with reduced out-of-focus background. The software can be used on series of at least two images acquired on available commercial microscopes equipped with a tunable pinhole, including confocal and stimulated emission depletion (STED) microscopes. We demonstrate applicability on different types of imaging modalities: (1) confocal imaging of DNA in a non-adherent cell line; (2) removal of out-of-focus background in super-resolved STED microscopy; (3) imaging of live intestinal organoids stained with a membrane dye.

## Introduction

In point-scanning microscopy, optical sectioning can be achieved using the pinhole, a small aperture placed in front of the detector^1^. The pinhole rejects the out-of-focus background, producing images of thin optical sections of the specimen. Pinhole-based detection is common in at least two important microscopy techniques, namely confocal microscopy and super-resolved stimulated emission depletion (STED) microscopy^2^. Pinhole-based confocal detection is employed by several quantitative fluorescence techniques such as Fluorescence Lifetime Imaging (FLIM)^3–5^, spectral imaging^6,7^, imaging of environment-sensitive dyes^8,9^, Fluorescence Recovery after Photobleaching (FRAP)^10^, Fluorescence Correlation Spectroscopy (FCS)^11,12^. Super-resolved STED microscopy is commonly implemented on a confocal architecture, and the pinhole is used to reject the out-of-focus background, especially in 2D STED microscopes^2^.

The maximum level of optical sectioning is theoretically obtained for the minimum size of the pinhole aperture, but this is normally prevented by the dramatic reduction of the detected signal when the pinhole is closed, leading to a compromise between axial resolution and signal-to-noise ratio (SNR)^13^. In practice, pinhole sizes significantly smaller than 1 AU can be used only with very bright specimens. The SNR limitation can be overcome by image scanning microscopy (ISM), a technique in which the pinhole plus detector is substituted by an array of multiple detector elements^14–16^. However, the ISM requires specialized confocal- or STED-ISM microscopes, not always available at imaging facilities. On the other hand, confocal and STED microscopes equipped with a tunable pinhole are more widely available, at least nowadays.

We have recently demonstrated that, in a confocal microscope, instead of working at closed pinhole, one can reach a similar level of optical sectioning by tuning the pinhole size and by analyzing the resulting image series^17^. The method, consists in the application of the separation of photons by lifetime tuning (SPLIT) algorithm to series of images acquired with tunable pinhole size (SPLIT-PIN)^17^. In SPLIT, the spatial resolution is improved by decoding the spatial information encoded into an additional channel of the microscope^18^. We have shown that this additional channel can be the fluorescence lifetime^18,19^, the depletion power^20,21^, the structured illumination pattern position^22^. In the SPLIT-PIN method, the additional channel is represented by size of the pinhole. In this channel, the signal originating from the in-focus region of the point spread function (PSF) has a different behavior than the signal coming from the out-of-focus region. Based on this, the out-of-focus background can be discarded by performing a linear decomposition in the phasor plot.

Here, we share and describe a user-friendly SPLIT-PIN software for the processing of series of images acquired at tunable pinhole size, which generates images with reduced out-of-focus background. The software can be used on series of at least two images acquired on available commercial microscopes equipped with a tunable pinhole. The software is based on the calculation of a modulation image, which encodes information on the axial position of the fluorescence emitters. Higher values of modulation correspond to a larger fraction of the out-of-focus component. At each pixel, the signal is decomposed into an in-focus fraction (component of low modulation) and an out-of-focus fraction (component of high modulation).

We demonstrate applicability on different types of imaging modalities where an efficient removal of out-of-focus background improves the quality of imaging: (1) confocal imaging of DNA in a non-adherent cell line; (2) removal of out-of-focus background in super-resolved stimulated emission depletion (STED) microscopy; (3) confocal imaging of live intestinal organoids stained with a membrane dye.

We evaluate the improvement provided by the SPLIT-PIN software using the recently introduced quality by image correlation spectroscopy (QuICS) algorithm^23^. QuICS quantifies the resolution, the contrast and the noise level in the images. In all cases QuICS reveals a significant increase of the image contrast in the final image.

In the Supplementary Text we provide detailed instructions for application of the software, even by non-expert users. We believe that this tool can be of interest for the many users of confocal and STED microscopes, given the wide availability of these technologies in research labs and imaging facilities.

## Results

### Description of the SPLIT-PIN software

**Figure 1:**
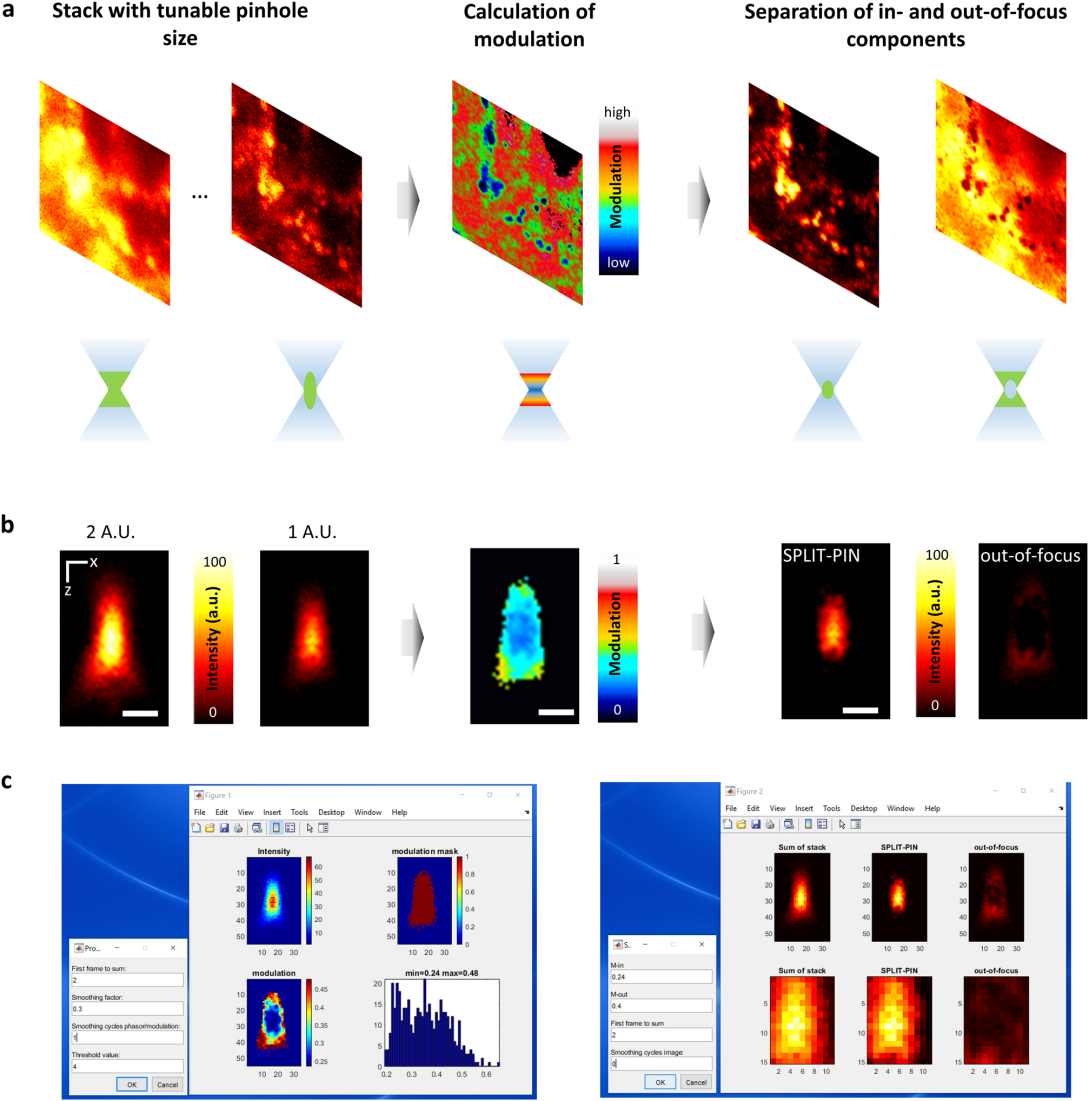
SPLIT-PIN algorithm. (**a**) (top) Workflow of the SPLIT-PIN approach: a series of images is acquired with a tunable pinhole size; a modulation image is calculated; the in-focus intensity is separated from the out-of-focus intensity. (bottom) Schematic representation of the effective detection volume corresponding to each of the image shown above. (**b**) Application of SPLIT-PIN to a series of confocal xz images of 200-nm fluorescent spheres acquired at the indicated pinhole size (A.U., Airy Units). Scale bar 500 nm. (**c**) Screenshots of the two main interactive menus in the user-friendly script, corresponding to the calculation of the modulation image (left) and to the generation of the SPLIT-PIN image (right), respectively.

The schematic workflow of the SPLIT-PIN method is reported in Fig. 1. A series of N images is acquired sequentially with a tunable pinhole size. This type of stack is typically obtained by a sequential acquisition and a minimum number N = 2 of images is required. The images are acquired with a decreasing pinhole size, so that the last image has the best optical sectioning. The image stack is then saved as a .TIF file. The SPLIT-PIN software is used to open the stack file and to calculate an intensity modulation image M(x,y) (See Methods for details on the calculation of modulation). The modulation image encodes additional spatial information: signal from fluorophores located in-focus has lower modulation (M_IN_), whereas signal from out-of-focus fluorophores has higher modulation (M_OUT_). In general, at each pixel, the fluorescence intensity will be the sum of the contribution from in-focus fluorophores (low modulation) and out-of-focus fluorophores (high modulation). The software uses the modulation image M(x,y) to calculate the fraction of the intensity originating from the in-focus fluorophores (f_IN_(x,y)) and separate it from the out-of-focus component (f_OUT_ = 1−f_IN_). The values M_IN_ and M_OUT_, required for the decomposition, are estimated directly from the data, as the 10% and 90% percentile values of the modulation histogram (Fig. 1c, left). The values M_IN_ and M_OUT_ are then used to generate the final SPLIT-PIN image (Fig. 1c, right). The final image is generated by multiplying the fraction f_IN_(x,y) by a total or partial sum of the images of the stack. The user can choose if using only the last image of the stack (best optical sectioning, worst SNR) or summing over all the images of the stack (best SNR, worst optical sectioning) (Fig. 1c, right).

In Fig. 1b the software is applied to a stack made of xz confocal images of 200-nm fluorescent spheres with pinhole size of 2 A.U. and 1 A.U. The modulation image clearly shows the difference of modulation between the in-focus and out-of-focus region. Application of the software generates a SPLIT-PIN image containing only the in-focus contribution, well separated from the out-of-focus component. Note that the SPLIT-PIN image provides better optical sectioning than the image acquired with 1 A.U., in other words we have ‘virtually’ closed the pinhole down to a smaller size.

### SPLIT-PIN imaging of DNA with virtually close pinhole

The first example shows the nucleus of a fixed U937-PR9 cell labeled with the DNA marker TO-PRO-3. U937 cells are a non-adherent cell line with an approximately spherical shape. More specifically, U937-PR9 cells are an in vitro model of oncogenic transformation^24,25^. Compared to adherent cells, where nuclei are often flattened along the lateral dimensions^26^, U937 cells have nuclei with approximately the same size in all the three dimensions. In other words, U937 nuclei do not appear squeezed along the optical axis. Thus, this system represents an ideal test to evaluate the improving of the optical sectioning performances provided by the SPLIT-PIN method in the cell nucleus. In this case, it was possible to perform imaging at 0.2 A.U., due to the high density of TO-PRO-3 labeling in the sample. Thus, tunable pinhole imaging was performed at 0.8 A.U. and 0.2 A.U. Note that 0.2 A.U. is the minimum value of pinhole size that can be set in the microscope acquisition software.

**Figure 2:**
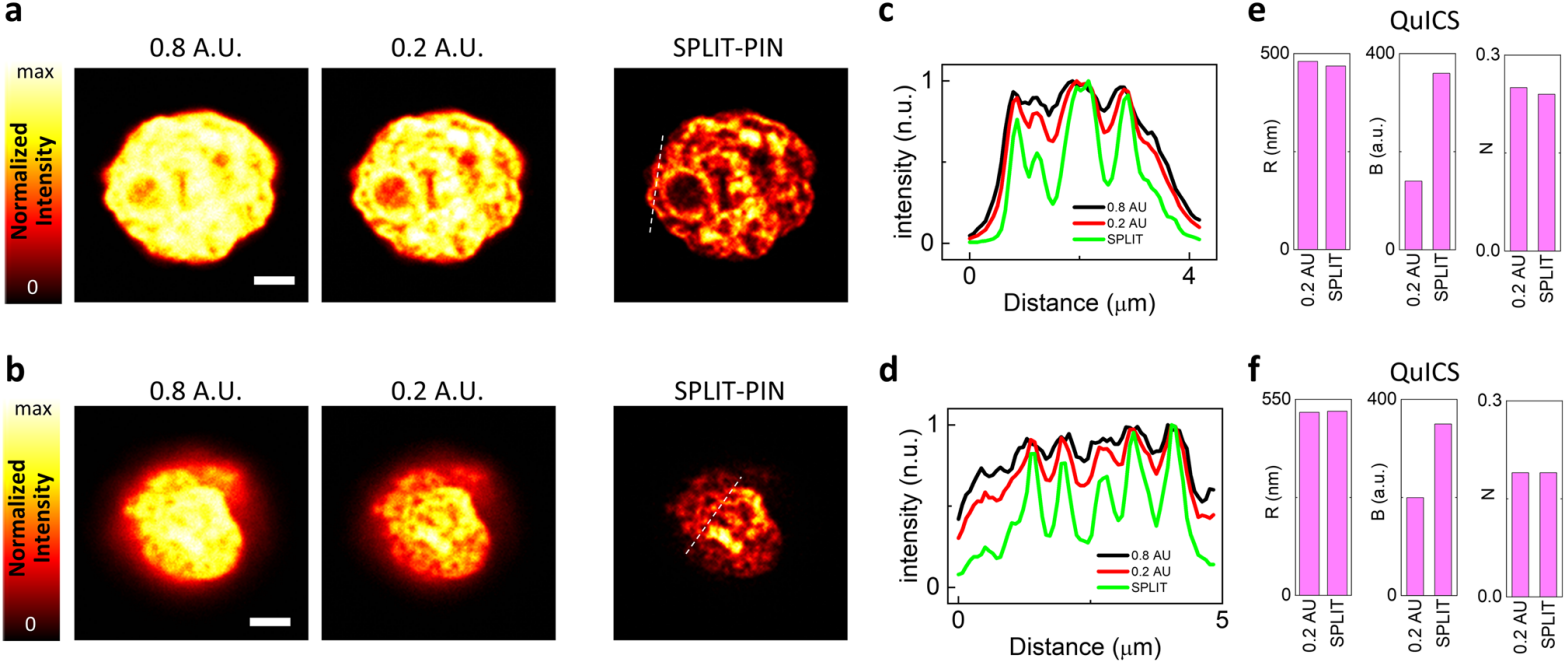
SPLIT-PIN imaging of DNA in non-adherent cell nuclei. (**a**, **b**) SPLIT-PIN imaging of fixed U937-PR9 cell nuclei labeled with the DNA dye To-Pro-3. Shown are confocal images acquired at 0.8 A.U. and 0.5. A.U. and the corresponding SPLIT-PIN images. Sections are shown at a mid plane (**a**) and at a bottom plane (**b**) of the nucleus. Scale bars 8 μm (**a**) and 2 μm (**b**). (**c**) Line profiles corresponding to the dashed line in (**a**). (**d**) Line profile corresponding to the dashed line in (**b**). (**e**) Quantification of Resolution (R), Brightness (B) and Noise (N) parameters for the SPLIT-PIN and 0.2 A.U. images shown in (**a**) using the QuICS algorithm. (**f**) Quantification of Resolution (R), Brightness (B) and Noise (N) parameters for the SPLIT-PIN and 0.2 A.U. images shown in (**b**) using the QuICS algorithm.

Figure 2a, b show series of two images acquired with 0.8 A.U. and 0.2 A.U pinhole size and the corresponding SPLIT-PIN images. Shown are optical sections at the equatorial plane (Fig. 2a) and at the bottom plane (Fig. 2b). In both cases, chromatin is visualized with higher contrast in the SPLIT-PIN image than in the 0.2 A.U. image, as shown by the line profiles (Fig. 2c, d). In the bottom section, it is possible to distinguish some holes in peripheral heterochromatin, probably corresponding to interchromatin compartment channels^27^. These regions of the interchromatin space are visualized with better contrast in the SPLIT-PIN image than in the 0.2 A.U. image. This is due to the increased optical sectioning capability of SPLIT-PIN.

To evaluate quantitatively the improvement provided by the SPLIT-PIN software, we applied the quality by image correlation spectroscopy (QuICS) algorithm^23^ to the images. QuICS is based on the calculation of a radial spatial autocorrelation function (ACF), i.e. a 2D spatial ACF that is angularly averaged. QuICS provides three parameters related to the quality of the image. The parameter R is related to the resolution of the optical system expressed as Full Width at Half Maximum (FWHM). More specifically, R ≥ FWHM, with the equal sign holding in the case of point-like objects. The parameter B represents the brightness, related to the image contrast. The parameter N represents the relative noise variance, related to the noise level in the image. For the equatorial section (Fig. 2e), QuICS reveals that the SPLIT-PIN image has comparable resolution (R = 468 nm), higher contrast (B = 360) and comparable noise level (N = 0.24) compared to the confocal image acquired at 0.2 A.U. (R = 480 nm, B = 140, N = 0.25). For the bottom section (Fig. 2f), QuICS reveals that the SPLIT-PIN image has comparable resolution (R = 517 nm), higher contrast (B = 350) and comparable noise level (N = 0.19) compared to the confocal image acquired at 0.2 A.U. (R = 514 nm, B = 200, N = 0.19). In both cases SPLIT-PIN provides a higher contrast compared to the image of smaller pinhole size.

### SPLIT-PIN removal of out-of-focus background in STED microscopy

**Figure 3:**
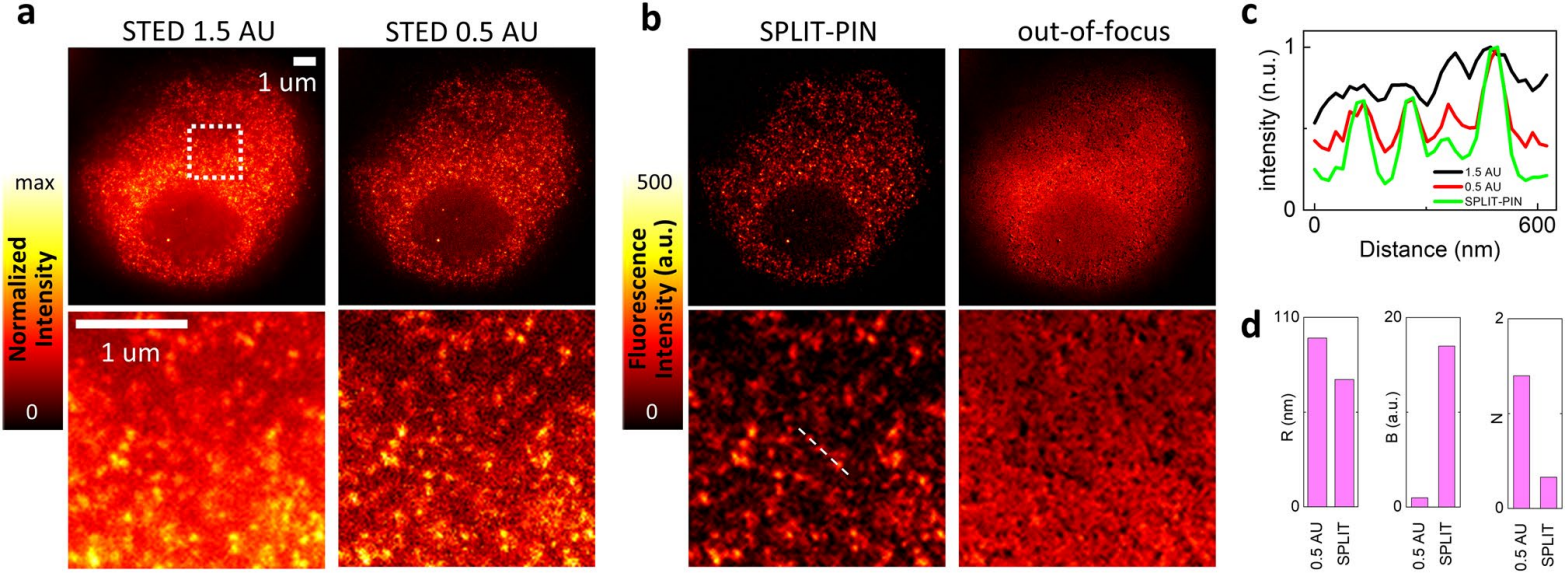
Removal of out-of-focus background in STED microscopy by SPLIT-PIN. (**a**) STED images acquired sequentially at different pinhole size. The images represent transcription foci in fixed U937-PR9 cells. b) SPLIT-PIN image (left) and isolated out-of-focus signal (right). Scale bar 1um. (**c**) Line profile corresponding to the dashed line in (**b**). (**d**) Quantification of Resolution (R), Brightness (B) and Noise (N) parameters for the SPLIT-PIN and 0.5 A.U. images shown in (**a**, **b**) using the QuICS algorithm.

In the second example we show how the SPLIT-PIN software can be applied to a super-resolution microscopy technique. Figure 3 shows a series of two stimulated emission depletion (STED) super-resolved images acquired sequentially with a different pinhole size. The image represents transcription foci in the nucleus of a fixed U937-PR9 cell. The images have been acquired with a 2D STED microscope providing improvement of resolution only in the lateral direction. We note that both STED images contains a background that reduces the contrast at which the transcription foci are visualized. The potential sources of background in STED microscopy and/or fluorescence correlation spectroscopy have been discussed in several papers^28–32^. Here we believe that the observed background signal is due to undepleted out-of-focus fluorescence signal. This hypothesis is confirmed by the fact that the regions of background have higher modulation value compared to the value of modulation at the foci (Supplementary Fig. 1). Application of SPLIT-PIN results in a STED image separated from the corresponding out-of-focus background (Fig. 3b). In the SPLIT-PIN image, transcription foci are visualized with better contrast, as shown by the line profile (Fig. 3c). More quantitatively, QuICS reveals that the SPLIT-PIN image has better resolution (R = 78 nm), higher contrast (B = 17) and lower noise level (N = 0.33) compared to the STED image acquired at 0.5 A.U (R = 98 nm, B = 0.97, N = 1.4). We note that the lateral resolution of the STED microscope is mainly determined by the STED beam power^20^ and should not depend on the pinhole size. Here, the variation of the parameter R is due to the almost 20-fold increase of contrast observed in the SPLIT-PIN image. This improvement of contrast results in a smaller size of the image correlation function and a smaller value of the parameter R.

### SPLIT-PIN imaging of live human intestinal organoids

**Figure 4:**
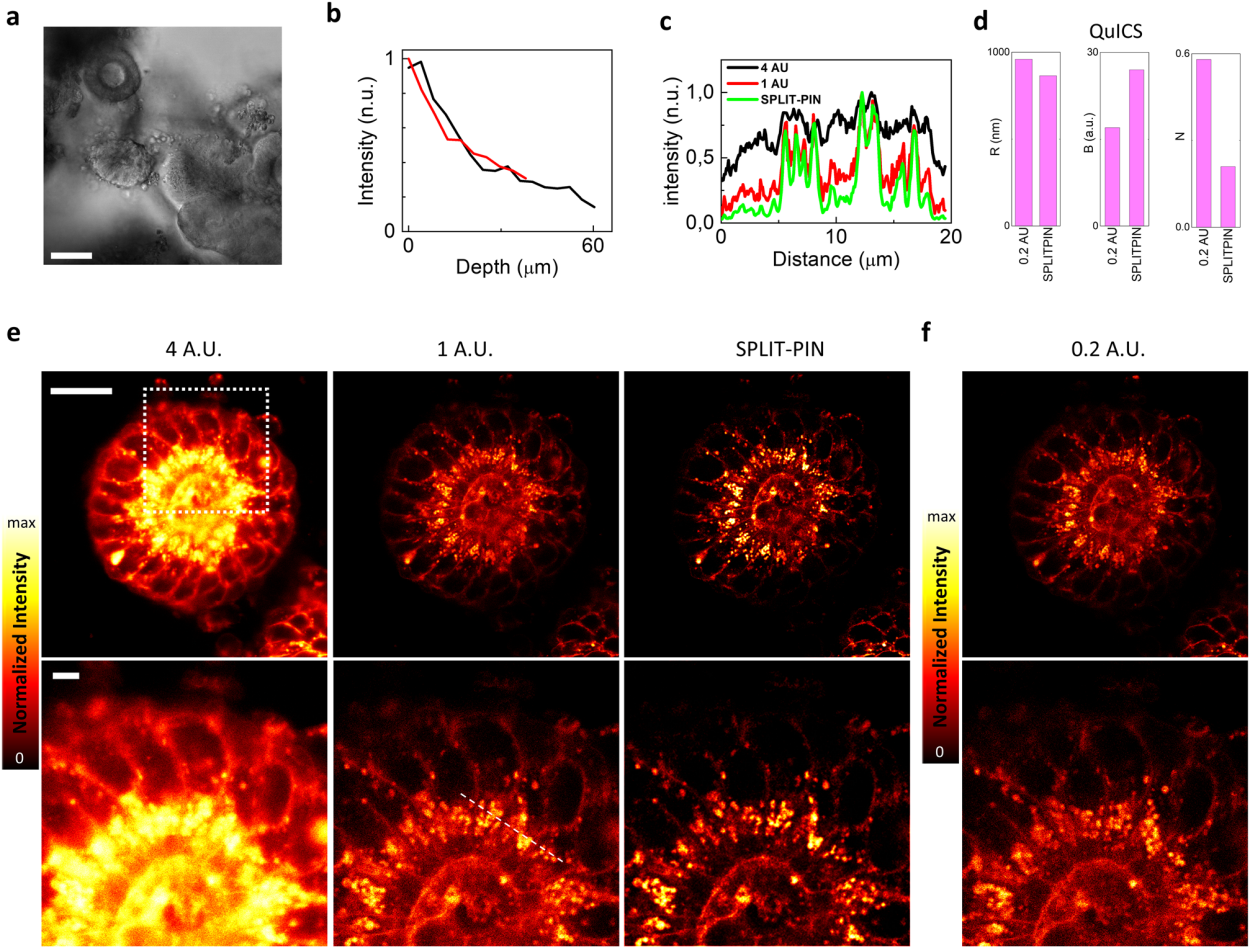
SPLIT-PIN imaging of live human intestinal organoids. (**a**) Bright field image of a sample of intestinal organoids. Scale bar 50 um. (**b**) Normalized fluorescence intensity as a function of the depth within the specimen. Data are obtained from two independent measurements. (**c**) Line profiles corresponding to the dashed line in (**e**). (**d**) Quantification of Resolution (R), Brightness (B) and Noise (N) parameters for the SPLIT-PIN and 0.2 A.U. images shown in (**e**) and (**f**) using the QuICS algorithm. (**e**) Series of confocal images of intestinal organoids labeled with CellMask Orange Plasma Membrane acquired with tunable pinhole size (4 A.U. and 1 A.U. respectively) and corresponding SPLIT-PIN image. Scale bar 20 um. (**f**) Confocal image of the same area of the specimen acquired at 0.2 A.U. and with an integration time equal to the total integration time of the confocal series shown in (**e**).

The third example shows application of SPLIT-PIN to confocal imaging of live intestinal organoids (Fig. 4a). Growing interest towards organoid models is related to their ability to mimic the cell-type composition and tissue organization of the native organ by recapitulating the self-organizing ability and the stem cell differentiation dynamic. Specifically, intestinal organoids derived from adult stem cells, isolated from human tissue biopsies, represent a more physiological in vitro model of the intestinal epithelium^33^, compared to conventional cell lines, such as CaCo-2 cells^34^. Thus, adult stem cell—derived organoids allow to observe direct interactions between different cell types, providing a reductionist approach yet retaining similarities with the in vivo tissue in cellular composition and tissue organization.

Organoids can be significantly thicker than cell monolayers. Confocal microscopy has limited imaging depth and the fluorescence intensity decreases as a function of the depth inside the sample. In our setup, at an imaging depth of about 40 um, the fluorescence intensity in the CellMask Orange channel decreases by a factor of about 3–4 (Fig. 4b). This limited photon budget makes imaging with a closed pinhole even more challenging.

Figure 4e shows application of SPLIT-PIN to confocal imaging of intestinal organoid (cystoid type) labeled with CellMask Orange Plasma Membrane. The dye stains the plasma membrane of the cells and other internal lipid components. A stack made of confocal images at 4 A.U. and 1 A.U. has been acquired using a resonant scanner. The use of a resonant scanner significantly speeds up the frame acquisition time, reducing the temporal lag between frames acquired sequentially with tunable pinhole size. The resulting SPLIT-PIN image has better contrast than the 4 A.U. and 1 A.U. images, as shown by the line profile (Fig. 4c). The SPLIT-PIN image is also compared with a confocal image acquired at 0.2 A.U. (0.2 A.U. is the minimum pinhole size available in the commercial setup) and with an integration time equal to the integration time of the confocal stack (Fig. 4f). QuICS analysis (Fig. 4d) reveals that the SPLIT-PIN image has better resolution (R = 865 nm), higher contrast (B = 27) and lower noise level (N = 0.21) compared to the image acquired at 0.2 A.U, with the same total integration time (R = 960 nm, B = 17, N = 0.58).

## Discussion

The main advantage of the SPLIT-PIN software is that it can be easily applied to stack of images acquired with microscopes equipped with a tunable pinhole. These include most confocal and STED microscopes, where a sequential acquisition with tunable pinhole size can be set up using the acquisition software. The simplest set of data, required for the generation of a SPLIT-PIN image, is a stack of N = 2 images acquired with a different pinhole size. Several image subtraction approaches have been proposed for the analysis of two images acquired with a different pinhole size^35–39^. In our opinion, there are some differences between the SPLIT-PIN method and the proposed image subtraction methods. The SPLIT-PIN image is not simply a linear combination of the original images of the stack, as shown by Eq. (11). The robustness of the non-linear filter depends on a proper choice of the parameters M_IN_ and M_OUT_. For this reason, these parameters are automatically estimated from the data but the user can also check and modify these values through a graphical user interface (GUI). The parameters M_IN_ and M_OUT_ correspond to the value of modulation for fluorophores which are in-focus and out-of-focus respectively, and the visualization of the modulation image is useful to avoid image processing artifacts. Another difference, compared to image subtraction methods, is that the SPLIT-PIN software can process stacks made of N > 2 images. Future investigations will be aimed at determining if SPLIT-PIN of series made of N > 2 frames can provide improvement of lateral resolution in confocal microscopy, in addition to the optical sectioning improvement.

We share a convenient, user-friendly script running under Matlab. Nevertheless, the image operations are extremely simple and can be easily implemented on any image analysis software (e.g. ImageJ). Given the versatility of confocal and STED microscopy and their widespread use in the biological sciences, we expect SPLIT-PIN to be easily integrated as a free tool to boost the optical sectioning power of the available laser scanning microscopes.

## Methods

### Cell culture and labeling

U937-PR9 cells were grown in Roswell Park Memorial Institute medium (RPMI-1640) medium (Sigma Aldrich R7388) added with 1% penicillin/streptomycin (Sigma-Aldrich P4333) and 10% fetal bovine serum (Sigma-Aldrich F9665) and maintained at 37 °C and 5% CO_2_. U937-PR9 were seeded on poly-l-lysine (Sigma-Aldrich P8920) coated glass coverslips before experiments. Cells were fixed with 4% (w/v) paraformaldehyde (PFA) at room temperature for 10 min. Next, cells were washed with 3% BSA and then permeabilized with 0.5% (v/v) Triton X-100 for 20 min.

For TO-PRO-3 staining, samples were extensively washed with PBS and stained with TO-PRO-3 (T3605-Thermo fisher scientific) at a dilution 1:1000 in BB, for 12 min at room temperature. For immunofluorescence experiments, cells were blocked with 3% BSA in PBS and incubated in a wet chamber with a RNA polymerase II CTD repeat (phospho S2) rabbit primary antibody (ab5095 Abcam) diluted in Incubation Buffer, overnight at 4 °C. Cells were then extensively washed with Washing Buffer 3 × 15 min and incubated with a Goat α-rabbit Atto 647N secondary antibody (40839 Sigma-Aldrich) diluted in IB for 1 h at room temperature, followed by the same washing procedure with WB. After washing with PBS, coverslips were mounted on glass slides with ProLong Diamond Antifade Mountant (Invitrogen P36961).

### Organoids preparation and labeling

Human samples were collected under study protocol approved by the Ethics Committee “Comitato Etico Catania 2” (Azienda Ospedaliera Garibaldi, Catania, prot. 601/C.E.) and informed consent was obtained from patients undergoing surgery for suspected colon adenocarcinoma. All methods were carried out in accordance with relevant guidelines and regulations. Healthy colon organoids were produced from patient-biopsy as previously described^40^ with minor modifications. Briefly, intestinal biopsy tissue was dissociated and washed in cold Phosphate Buffer Saline without Ca^++^ and Mg^++^ (Euroclone) supplemented with 50 mg/ml gentamicin (Life Technologies), P/S 100U (Euroclone) and 2.5 mg/ml amphotericin (Life Technologies) in order to prevent common contaminations. On a rocking platform, colon fragments were treated with Gentle Dissociation Reagent (STEMCELL Technologies™, Inc. (STI)) on ice for 30 min. Then, the fragments were allowed to settle by gravity, resuspended in DMEM F12 with 15 mM HEPES (STEMCELL Technologies Inc. (STI) supplemented with BSA (Sigma) at 1% in Phosphate Buffer Saline without Ca^++^ and Mg^++^ (Euroclone) and passed through 70 μm cell strainers. The crypts obtained were resuspended in a 1:1 mixture of DMEM F12 with 15 mM HEPES ((STEMCELL Technologies Inc. (STI)) + 1% BSA and Matrigel® (Corning) and 50 μl/well of the suspension was pipetted into pre-warmed 24-well TC plates (Euroclone) to form domes that were solidified at 37 °C for 15 min. At the end of this time, 750 μl/well of complete IntestiCult™ Organoid Growth Medium (STEMCELL Technologies Inc. (STI) was added and organoids were maintained in incubator at controlled temperature (37 °C) and CO_2_ (5%). In order to perform organoids maintenance, the medium was changed 2–3 times per week and organoids were passaged every 4–7 days.

In order to prepare organoids for live-confocal imaging, the medium was removed and organoids were recovered from the Matrigel® incubating on ice and on a rocking platform for 20 min with GCDR. Once the organoids are settled by gravity, the supernatant was aspirated and organoids were ready for the labeling step. Labeling was performed at 37 °C with CellMask Orange Plasma Membrane stain (Thermofisher C10045) at a dilution 1:1000 respectively for 60 min at 37 °C. Before imaging, organoids were transferred into on 8-well chambered coverslips (µ-Slide 8 Well Glass Bottom, ibidi 80827, Germany).

### Image acquisition

Series of multiple confocal or STED images at different pinhole size were acquired using the frame-sequential acquisition^17^. The values of pinhole size were set as specified. The excitation power was kept constant unless specified otherwise. The number of line averaging was kept constant unless specified otherwise.

Confocal images were acquired on a Leica TCS SP8 confocal microscope, using an HCX PL APO CS2 63 × 1.40 NA oil immersion objective lens (Leica Microsystems, Mannheim, Germany). Tetraspeck fluorescent spheres with a size of 200 nm (TetraSpeck Fluorescent Microspheres Size Kit, ThermoFisher) were excited at 488 nm and their fluorescence emission was detected at 500–550 nm. TO-PRO-3 was excited at 633 nm and its fluorescence emission was detected at 640–700 nm using a hybrid detector (Leica Microsystems). CellMask Orange was excited at 561 nm and its fluorescence emission was detected at 565–650 nm using a hybrid detector (Leica Microsystems).

STED images were acquired on a Leica Stellaris 8 Tau-STED microscope, using an HC PL APO CS2 100 × 1.40 NA oil immersion objective lens (Leica Microsystems, Mannheim, Germany). Stimulated emission depletion was accomplished with a 775 nm STED laser. A white light laser provided excitation at the desired wavelength for each sample. Excitation wavelengths/emission bandwidths were the following: Atto647N (646, 651-720). 1024 × 1024 pixel images were acquired with a pixel size of 19 nm.

### SPLIT-PIN algorithm

For a given image stack *I*_*j*_*(x,y)* the images are processed with the phasor analysis in which variables $g\left(x,y\right)$ and $s(x,y)$ are calculated as:

1 $$g\left(x,y\right)=\frac{\sum_{j=1 }^{N}{I}_{j}(x,y)\mathrm{cos}\left[\frac{2\pi \left(j-1\right)}{N}\right]}{\sum_{j=1 }^{N}{I}_{j}(x,y)}$$

2 $$s\left(x,y\right)=\frac{\sum_{j=1 }^{N}{I}_{j}(x,y)\mathrm{sin}\left[\frac{2\pi \left(j-1\right)}{N}\right]}{\sum_{j=1 }^{N}{I}_{j}(x,y)}$$

where *N* is the number of the images of the stack.

The modulation $M\left(x,y\right)$ is calculated as:

3 $$M\left(x,y\right)={\left({g}^{2}\left(x,y\right)+{s}^{2}\left(x,y\right)\right)}^{1/2}$$

The value of modulation at each pixel, *M*(x,y), can be expressed as a linear combination of of 2 components:

4 $$M\left(x,y\right)={f}_{IN}\left(x,y\right){M}_{IN}+\left({1-f}_{IN}\left(x,y\right)\right){M}_{OUT}$$

where M_IN_ represents the value of modulation for fluorophores located at the center of the focal spot (i.e. in focus), M_OUT_ represents the value of modulation at the periphery of the focal spot (i.e. out of focus) and *f*_*IN*_(x,y) represents the fraction of the intensity at pixel (x,y) originating from the center of the focal spot. For each pixel, linear inversion of Eq. (4) leads to the fraction ${f}_{IN,LIN}(x,y)$, calculated as:

5 $${f}_{IN,LIN}\left(x,y\right)=1-\frac{M\left(x,y\right)-{M}_{IN}}{{M}_{OUT}-{M}_{IN}}$$

To force values of fraction to fall between 0 and 1, the values of f_IN,LIN_(x,y) are filtered through a logistic function of the form:

6 $${f}_{IN}\left(x,y\right)=1/\left(1+{e}^{-{k}_{L}\left({f}_{IN,LIN}\left(x,y\right)-1/2\right)}\right)$$

with k_L_ = 4, as described previously^22^.

Finally, the SPLIT-PIN image and the out-of-focus component are calculated as:

7 $${I}_{SPLIT-PIN}\left(x,y\right)={f}_{IN}\left(x,y\right){I}_{SUM}\left(x,y\right)$$

8 $${I}_{out-of-focus}\left(x,y\right)=\left(1-{f}_{IN}\left(x,y\right)\right){I}_{SUM}\left(x,y\right)$$

The image I_sum_ represents a total or partial sum of images of the stack:

9 $${I}_{SUM}\left(x,y\right)=\sum_{j={j}_{0} }^{N}{I}_{j}(x,y)$$

The value j_0_ is the first frame to be summed in the stack. Assuming that images are acquired with a decreasing pinhole size, j_0_ = 1 corresponds to the image with largest pinhole size and j_0_ = N corresponds to the image with smallest pinhole size. For instance, if one has a good signal-to-noise ratio in the last image of the stack, one can set j_0_ = N. In this case, one maximizes the resolution of the SPLIT-PIN image. On the contrary, if the signal-to-noise ratio in the last frame of the stack is poor, one can use a value j_0_ < N and increase the SNR of both the sum image and the SPLIT-PIN image.

The values of M_IN_ and M_OUT_ are directly estimated from the modulation histogram. The modulation histogram was built as the histogram of the values M(x,y) corresponding to pixels with intensity above a given threshold (selected by the user). The values of M_IN_ and M_OUT_ were calculated as the 10% and 90% percentile values of the histogram, respectively.

A user-friendly version of the Matlab (The MathWorks) code is available at https://github.com/llanzano/SPLITPIN. A step-by-step description of this user-friendly version is available as Supplementary Text.

### Generation of the SPLIT-PIN image from stacks of two images

For the specific case N = 2, one of the two phasor components is systematically null, s(x,y) = 0. In this case, the calculation of modulation is simplified and given by the following:

10 $$M\left(x,y\right)=\frac{{F}_{1}\left(x,y\right)-{F}_{2}(x,y)}{{F}_{1}\left(x,y\right)+{F}_{2}(x,y)}=\frac{{I}_{DIF}\left(x,y\right)}{{I}_{SUM}(x,y)}$$

In other words, the modulation is the ratio between the difference of the two images I_DIF_(x,y) and their sum I_SUM_(x,y). It follows from Eqs. (5)–(10) that:

11 $${I}_{SPLIT-PIN,N=2}\left(x,y\right)={\left(1+{e}^{-{k}_{L}\left(\frac{1}{2}-\frac{\frac{{I}_{DIF}\left(x,y\right)}{{I}_{SUM}\left(x,y\right)}-{M}_{IN}}{{M}_{OUT}-{M}_{IN}}\right)}\right)}^{-1}{I}_{SUM}\left(x,y\right)$$

Equation (11) shows that, in the case of N = 2, the final SPLIT-PIN image is not simply the linear combination of the two images of the stack. The non-linearity is introduced by the non-linear filter in Eq. (6).

### Image analysis

The quality of the generated SPLIT-PIN images was analyzed the Quality by Image Correlation Spectroscopy (QuICS) algorithm^23^. The QuICS analysis was performed in MATLAB using the code available at https://github.com/llanzano/QuICS. Briefly, given an image I(x,y), a two-dimensional (2D) image correlation function *G*_2D_(*δ*_x_,*δ*_y_) was calculated as:

12 $${G}_{2D}\left({\delta }_{x},{\delta }_{y}\right)=\frac{\langle I\left(x,y\right)I\left(x+{\delta }_{x},y+{\delta }_{y}\right)\rangle }{{\langle I\left(x,y\right)\rangle }^{2}}-1$$

where *δ*_x_ and *δ*_y_ are the spatial lag variables, I(x,y) is the fluorescence intensity detected at pixel.

(x,y), the angle brackets indicate averaging over all the selected pixels of the image. The numerator in Eq. (5) was calculated by a 2D fast Fourier transform algorithm. The radial correlation function G(*δ*_r_) was calculated by performing an angular mean^41^. The noise-free correlation function was estimated by performing a Gaussian fit of the correlation function G(*δ*) by skipping the zero lag point:

13 $$G_{NF} \left( \delta \right) = G_{NF} \left( 0 \right)e^{{ - \frac{{\delta^{2} }}{{w^{2} }}}} + G_{NF} \left( \infty \right)\;\delta \in \left[ {1,\delta_{max} } \right]$$

where the width parameter *w* corresponds to the 1/e^2^ of a Gaussian function and it is related to the Full Width Half Maximum (FWHM) by the relationship w = FWHM/(2ln2)^1/2^; G_NF_(0) represents the amplitude; G_NF_(∞) represents an offset value. The value $\delta$_max_ was determined so as to fit a single Gaussian component. The parameters R, B, N have been calculated as:

14 $$R=\sqrt{2ln2}w$$

15 $$B={G}_{NF}\left(0\right){I}_{av}$$

16 $$N=\frac{G\left(0\right){-G}_{NF}\left(0\right)}{{G}_{NF}\left(0\right)}$$

where we have indicated I_av_ as the average intensity value over all the pixels of the image. R is the width of the autocorrelation function, related to the spatial resolution; B is the brightness, related to the image contrast; N is the relative noise variance, related to the signal-to-noise ratio of the image^23^.

## Supplementary Information

Supplementary Information.

## Data Availability

The datasets used and/or analyzed during the current study are available from the corresponding author on reasonable request.

## References

[1] Diaspro A, Bianchini P (2020). Optical nanoscopy. La Riv. Nuovo Cimen..

[2] Vicidomini G, Bianchini P, Diaspro A (2018). STED super-resolved microscopy. Nat. Methods.

[3] Day RN (2014). Measuring protein interactions using Forster resonance energy transfer and fluorescence lifetime imaging microscopy. Methods.

[4] Pelicci S, Diaspro A, Lanzano L (2019). Chromatin nanoscale compaction in live cells visualized by acceptor-to-donor ratio corrected Forster resonance energy transfer between DNA dyes. J. Biophoton..

[5] Broussard JA, Rappaz B, Webb DJ, Brown CM (2013). Fluorescence resonance energy transfer microscopy as demonstrated by measuring the activation of the serine/threonine kinase Akt. Nat. Protoc..

[6] Fereidouni F, Bader AN, Gerritsen HC (2012). Spectral phasor analysis allows rapid and reliable unmixing of fluorescence microscopy spectral images. Opt. Express.

[7] Shi W (2020). Pre-processing visualization of hyperspectral fluorescent data with spectrally encoded enhanced representations. Nat. Commun..

[8] Malacrida L (1858). Spectral phasor analysis of LAURDAN fluorescence in live A549 lung cells to study the hydration and time evolution of intracellular lamellar body-like structures. Biochem. Biophys. Acta..

[9] Sediqi H, Wray A, Jones C, Jones M (2018). Application of spectral phasor analysis to sodium microenvironments in myoblast progenitor cells. PLoS ONE.

[10] Fritzsche M, Charras G (2015). Dissecting protein reaction dynamics in living cells by fluorescence recovery after photobleaching. Nat. Protoc..

[11] Di Bona M (2019). Measuring mobility in chromatin by intensity-sorted FCS. Biophys. J..

[12] Scipioni L, Di Bona M, Vicidomini G, Diaspro A, Lanzanò L (2018). Local raster image correlation spectroscopy generates high-resolution intracellular diffusion maps. Commun. Biol..

[13] Sheppard, C. J. R. G. X., Gu, M. & Roy, M. In *Handbook of Biological Confocal Microscopy* (ed Pawley, J. B.) (Springer, 2006).

[14] Gregor I, Enderlein J (2019). Image scanning microscopy. Curr. Opin. Chem. Biol..

[15] Castello M (2019). A robust and versatile platform for image scanning microscopy enabling super-resolution FLIM. Nat. Methods.

[16] Korobchevskaya KLBC, Colin-York H, Fritzsche M (2017). Exploring the potential of Airyscan microscopy for live cell imaging. Photonics.

[17] D'Amico M (2022). A phasor-based approach to improve optical sectioning in any confocal microscope with a tunable pinhole. Microsc. Res. Tech..

[18] Lanzano L (2015). Encoding and decoding spatio-temporal information for super-resolution microscopy. Nat. Commun..

[19] Tortarolo G (2019). Photon-separation to enhance the spatial resolution of pulsed STED microscopy. Nanoscale.

[20] Sarmento MJ (2018). Exploiting the tunability of stimulated emission depletion microscopy for super-resolution imaging of nuclear structures. Nat. Commun..

[21] Pelicci S, Tortarolo G, Vicidomini G, Diaspro A, Lanzanò L (2020). Improving SPLIT-STED super-resolution imaging with tunable depletion and excitation power. J. Phys D Appl. Phys..

[22] Cainero I (2021). Chromatin investigation in the nucleus using a phasor approach to structured illumination microscopy. Biophys. J..

[23] Cerutti E (2021). Evaluation of sted super-resolution image quality by image correlation spectroscopy (QuICS). Sci. Rep..

[24] Cainero I (2010). Measuring nanoscale distances by structured illumination microscopy and image cross-correlation spectroscopy (SIM-ICCS). Sens. (Basel).

[25] Cerutti E (2022). Alterations induced by the PML-RARalpha oncogene revealed by image cross-correlation spectroscopy. Biophys. J..

[26] Li Y (2015). Moving cell boundaries drive nuclear shaping during cell spreading. Biophys. J..

[27] Cremer T (2020). The interchromatin compartment participates in the structural and functional organization of the cell nucleus. BioEssays.

[28] Ringemann C (2009). Exploring single-molecule dynamics with fluorescence nanoscopy. New J. Phys..

[29] Coto-Hernandez I (2014). A new filtering technique for removing anti-Stokes emission background in gated CW-STED microscopy. J. Biophoton..

[30] Lanzano L (2017). Measurement of nanoscale three-dimensional diffusion in the interior of living cells by STED-FCS. Nat. Commun..

[31] Gao P, Prunsche B, Zhou L, Nienhaus K, Nienhaus GU (2017). Background suppression in fluorescence nanoscopy with stimulated emission double depletion. Nat. Photon..

[32] Ma Y, Ha T (2019). Fight against background noise in stimulated emission depletion nanoscopy. Phys. Biol..

[33] Nikolaev M (2020). Homeostatic mini-intestines through scaffold-guided organoid morphogenesis. Nature.

[34] Giral H (2012). NHE3 regulatory factor 1 (NHERF1) modulates intestinal sodium-dependent phosphate transporter (NaPi-2b) expression in apical microvilli. J. Biol. Chem..

[35] Heintzmann R (2003). Resolution enhancement by subtraction of confocal signals taken at different pinhole sizes. Micron.

[36] Kakade R, Walker JG, Phillips AJ (2015). Optimising performance of a confocal fluorescence microscope with a differential pinhole. Meas. Sci. Technol..

[37] Martinez-Corral M, Caballero MT, Ibanez-Lopez C, Sarafis V (2003). Optical sectioning by two-pinhole confocal fluorescence microscopy. Micron.

[38] Okugawa, H. *A New Imaging Method for Confocal Microscopy,* Vol. 6860 PWB (SPIE, 2008).

[39] Wang Y, Kuang C, Gu Z, Liu X (2013). Image subtraction method for improving lateral resolution and SNR in confocal microscopy. Opt. Laser Technol..

[40] Sato T (2011). Long-term expansion of epithelial organoids from human colon, adenoma, adenocarcinoma, and Barrett's epithelium. Gastroenterology.

[41] Scipioni L, Gratton E, Diaspro A, Lanzanò L (2016). Phasor analysis of local ICS detects heterogeneity in size and number of intracellular vesicles. Biophys. J..

