# Supplementary material for: SPLIT-PIN software enabling confocal and super-resolution imaging with a virtually closed pinhole
Source: Sci Rep. 2023 Feb 15;13:2741. doi: 10.1038/s41598-023-29951-9 (PMC9931717; doi:10.1038/s41598-023-29951-9)
Supplement: Supplementary file 1 — Supplementary Information. [file 41598_2023_29951_MOESM1_ESM.pdf]

Supporting information for

SPLIT-PIN software enabling confocal and super-resolution imaging with a virtually closed pinhole

Figure S1

Supplementary Text - Description of the SPLIT-PIN software

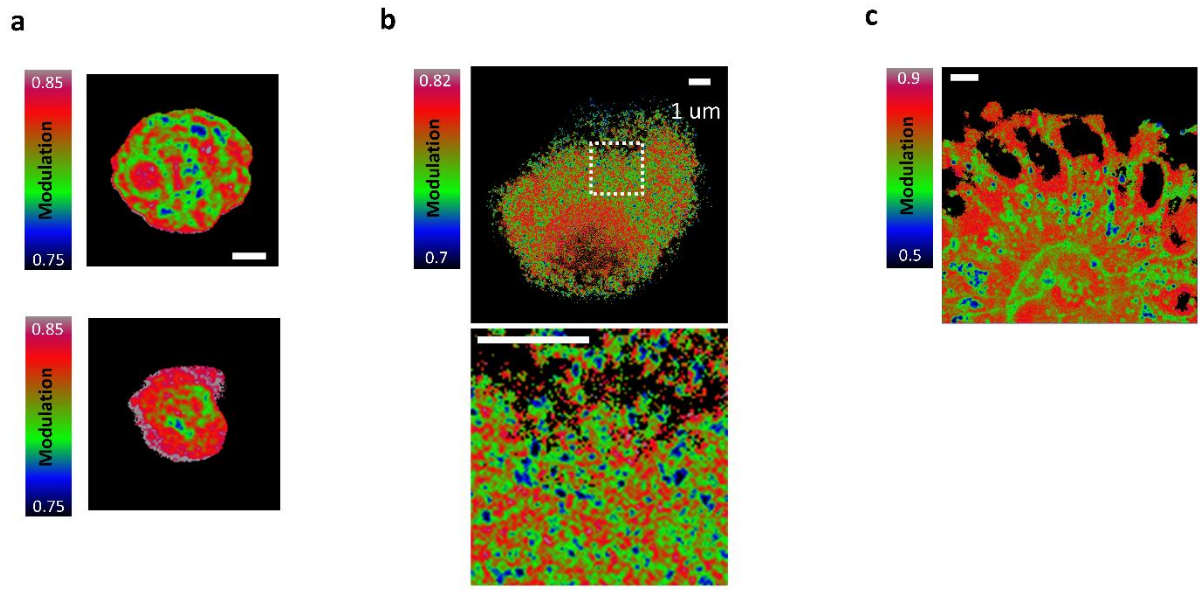

Figure S1. Modulation images.

(a-c) Modulation images corresponding to the data shown in Fig.2 (a), Fig.3 (b), Fig.4 (c) of the main text. Scale bars 2 um (a), 1 um (b), 20 um (c).

Supplementary text - Description of the SPLIT-PIN software

Go to https://github.com/llanzano/SPLITPIN and download the file: simpleSPLIT-PIN_v1.m.

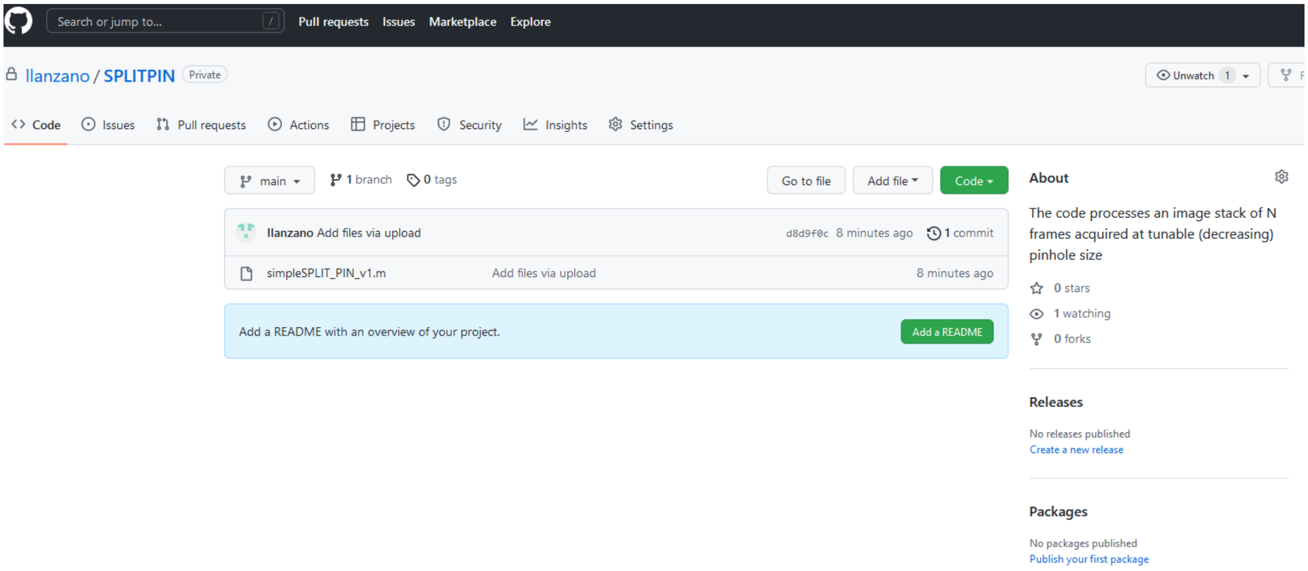

Figure 1 Download the software on github.com

For example, we want to run the script "simpleSPLIT-PIN_v1.m". First, download the script in any folder in your PC (it does not need to be in the MATLAB folder). Now, you can run MATLAB with a double click on the icon, or you can double click on the script you downloaded. Once MATLAB is open, you will have this interface:

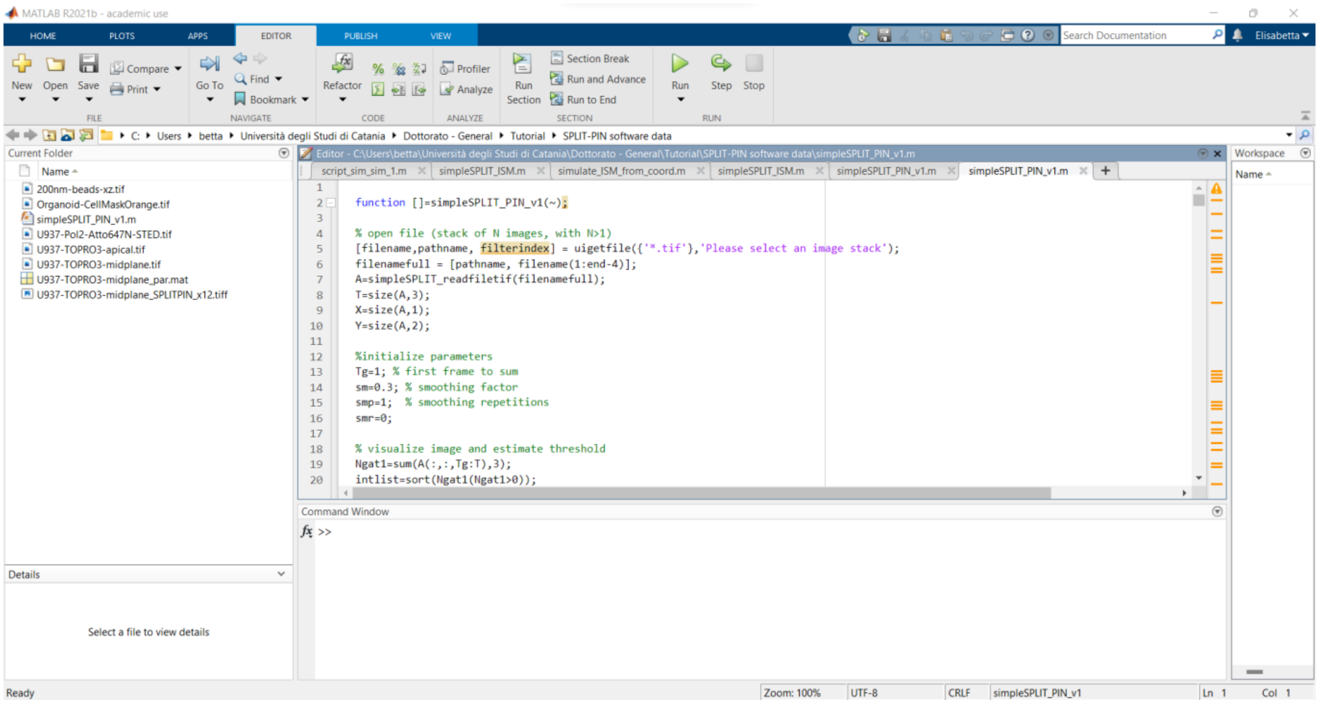

Figure 1 MatLab Interface

In Figure 2 it is possible to see the script simpleSPLIT_PIN_v1.m is opened. On the left side of the interface, you can see your data, you MatCode and a file .mat that contains some parameters.

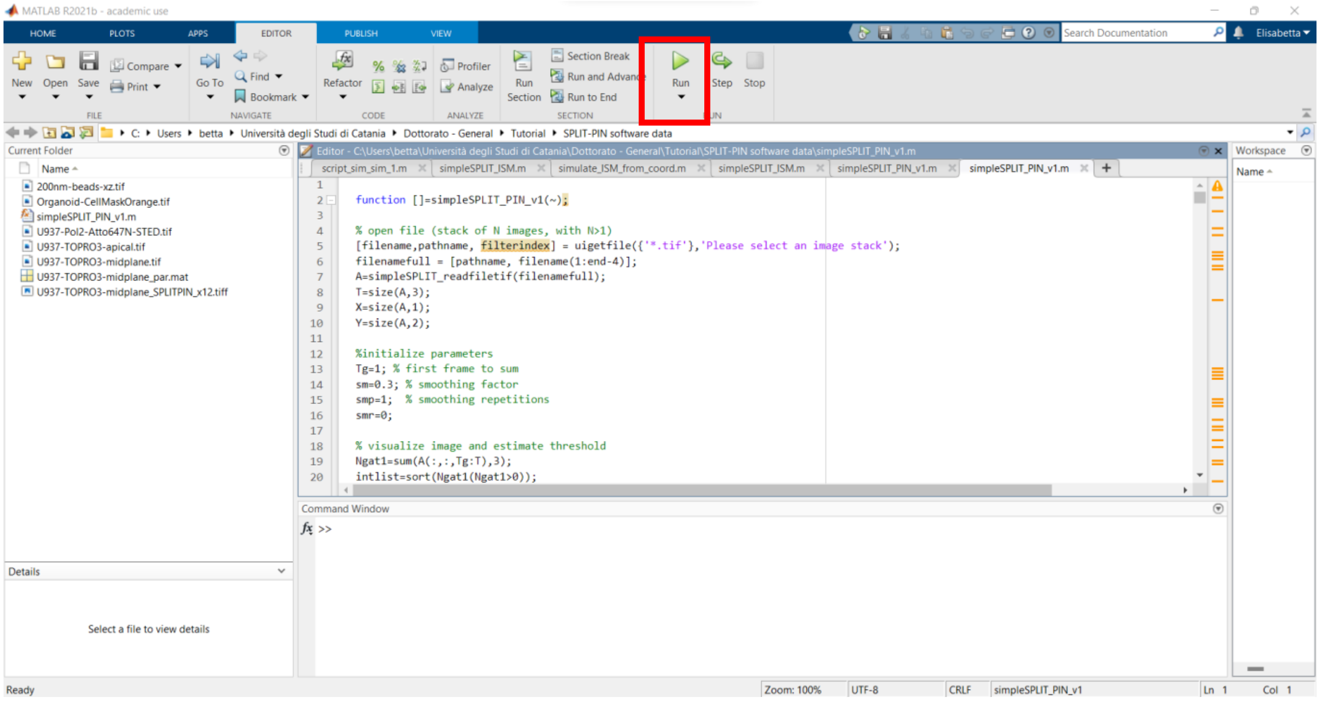

Figure 2 Run the script

To Run the script, you have click on the button “RUN” as you can see in Figure 3. Once you click on Run, MATLAB will open a window in which there are your files. The script opens a .TIF image stack. The stack is a series of 2 or more images acquired with tunable pinhole size. You must select (with a double click on the file or selecting the file and then click on “Open”) the .TIF stack you want to process (Figure 4). For example, open the stack U937-TOPRO3-midplane.

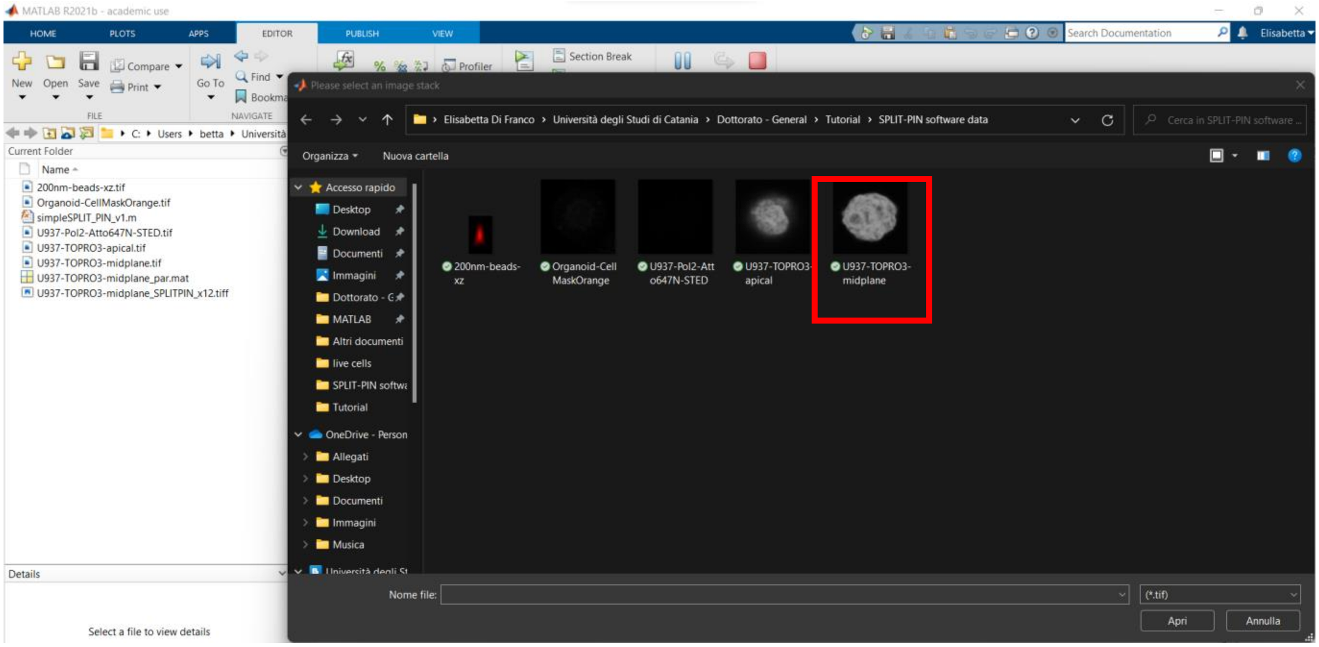

Figure 3 Selection of TIF image

Before being able to start processing the image, it is possible to select a .MAT file that allows you to load the same processing parameters used for a previous analysis (Figure 5). If you don't have such a file or you don't want to use previously used parameters, press cancel.

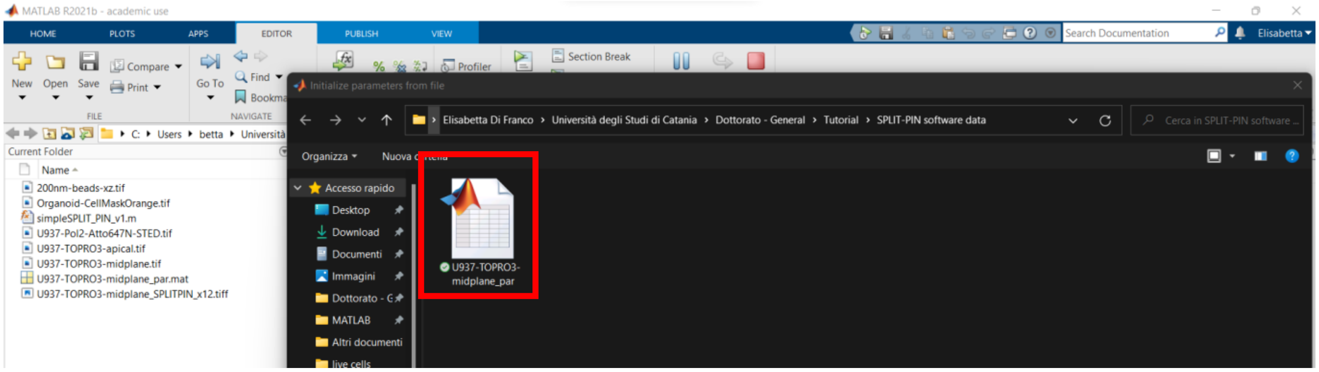

Figure 4 Selection of the file that contains the processing parameters

After this step, the script calculates the modulation image:

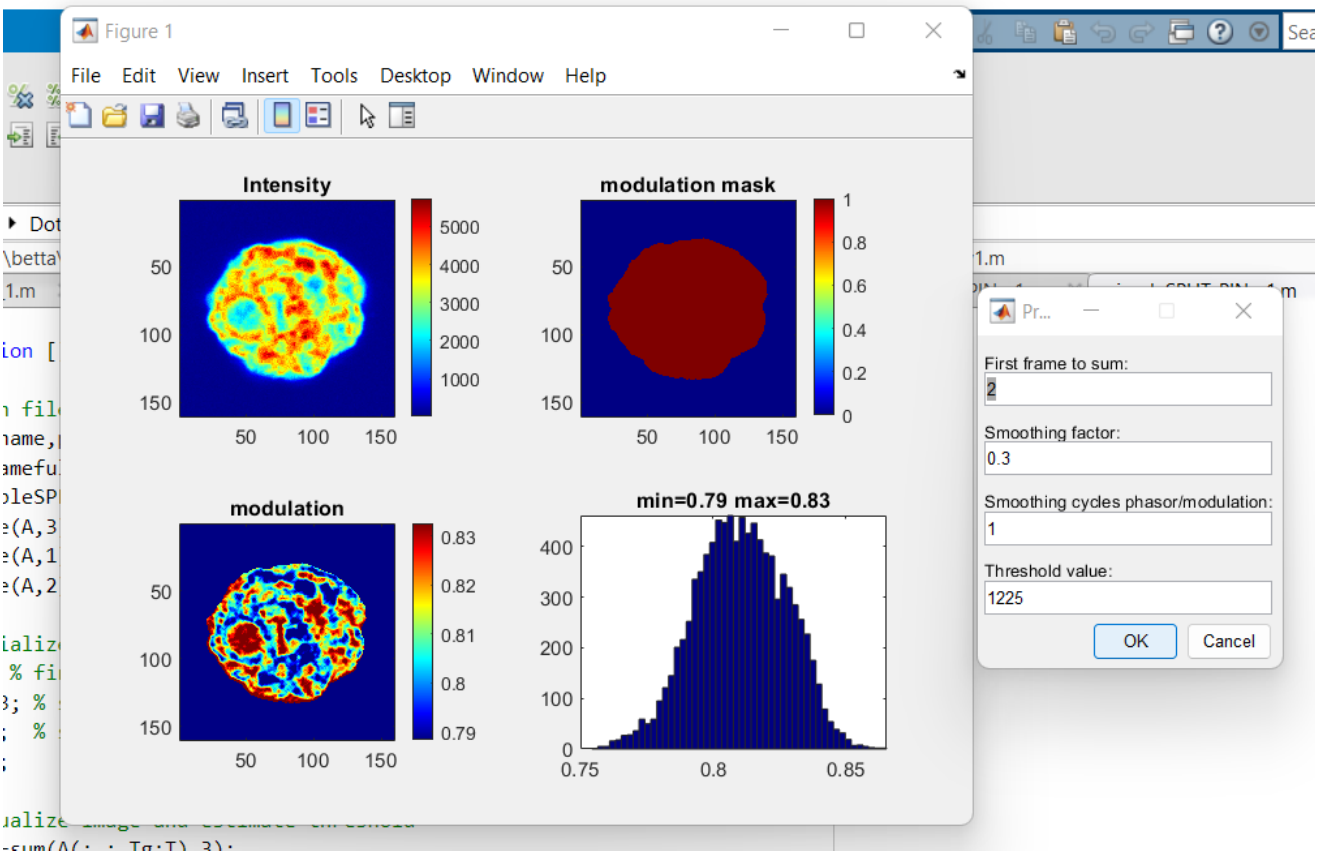

Figure 5 Calculation of the modulation image

In Figure 6 you will see the window with the 4 subplots and a menu. In this menu, the parameters are prefilled with default values or with the values loaded from a parameter file. These parameters are:

- First Frame to sum: you can choose from which frame you want to start the sum of the stack to generate the intensity image. In the example, the stack is composed of two frames, so we can choose 1 or 2. Default value=2.
- Smoothing factor: to smooth the modulation image in your image. Values from 0 (no smoothing) to 1 (maximum smoothing); default value=0.3.
- Smoothing cycles: number of cycles to repeat the smoothing process; default value=1.
- Threshold value: select the threshold value in such a way that the modulation mask correspond to your region of interest (i.e. exclude regions of background). The threshold is used for visualization of the modulation image and for calculation of the histogram. Default value: median of the intensity values of the image. In Figure 6, this parameter is automatically set to the value of 1225.

You can change the parameters to visualize better the modulation image. Press OK to apply the new values. Press Cancel to proceed to next step.

The final step is the generation of the SPLIT-PIN image:

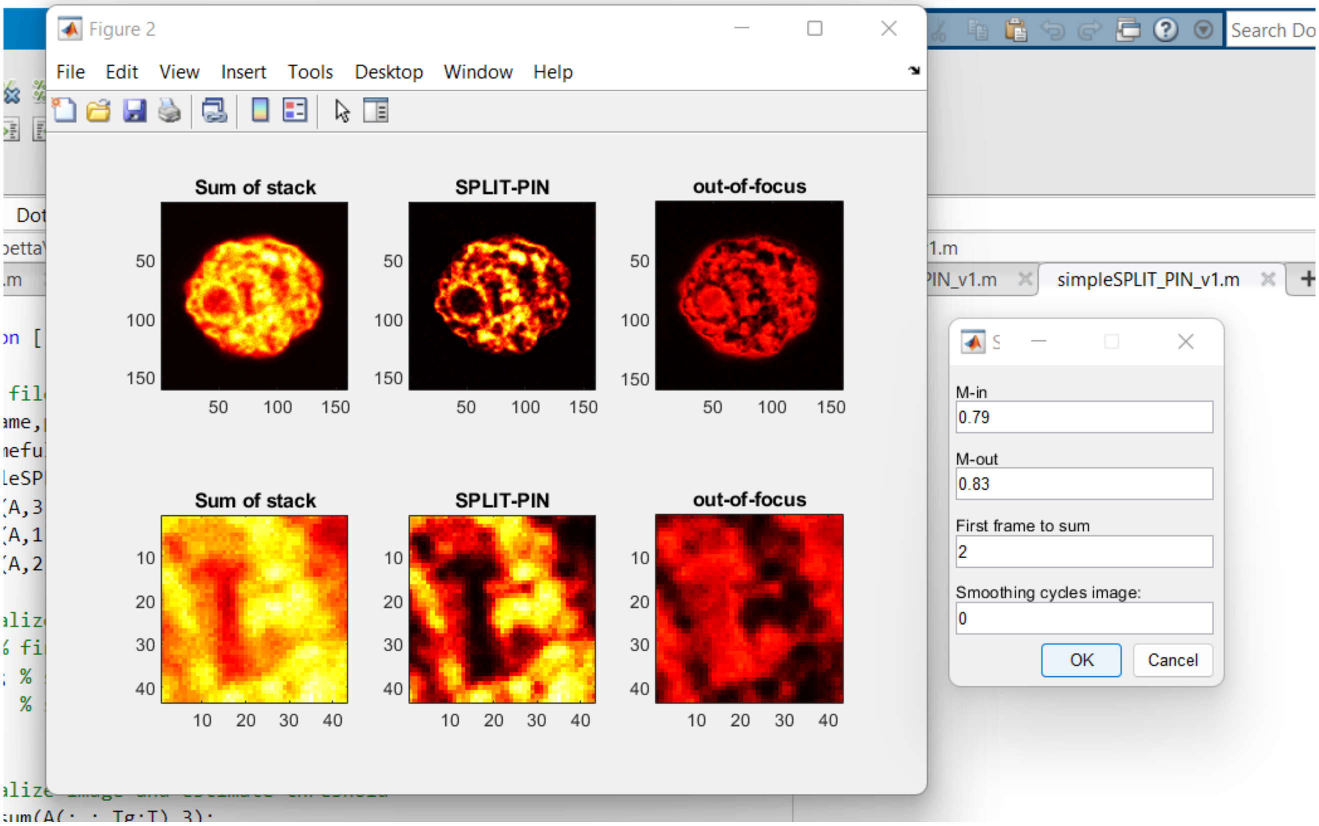

Figure 7 SPLIT images

In Figure 7, it is possible to see the SPLIT-PIN image separated from the out-of-focus component. Also in this case, there is a menu:

- M-in: modulation corresponding to the in-focus component. Default value=value estimated from the histogram
- M-out: modulation corresponding to the out-of-focus component. Default value=value estimated from the histogram
- First Frame to sum: you can choose from which frame you want to start to generate the 'sum of stack' image. In the example, the stack is composed of two frames, so we can choose 1 or 2. Default value=2.
- Smoothing cycles image: number of cycles to apply the smoothing process to the 'Sum of stack' image; default value=0.

You can change the parameters to optimize the final image. Press OK to apply the new values. Press Cancel to proceed to finish the processing.

The final window asks you if you want to save your data. Press OK to save the data.

- The SPLIT-PIN image is saved as 16-bit .TIFF file. The intensity values of the SPLIT-PIN image are multiplied by a factor k to use the full dynamic range of the 16-bit image. The value of the multiplying factor is reported in the name of the file. For instance, in our example the file is saved as: U937-TOPRO3-midplane_SPLITPIN_x12.tiff (i.e. the values have been multiplied by a factor of 12).
- The Modulation image is saved as 16-bit .TIFF file. The values of the Modulation image are multiplied by a 100 to use the full dynamic range of the 16-bit image. The value of the multiplying factor is reported in the name of the file. For instance, in our example the file is saved as: U937-TOPRO3-midplane_mod_x100.tiff.
- The processing parameters are saved as a .mat file with name "filename_par.mat" (in our example, U937-TOPRO3- midplane_par.mat)
